# Optimization of the Use of Industrial Wastes in Anaerobic Soil Disinfestation for the Control of Fusarium Wilt in Strawberry

**DOI:** 10.3390/plants12183185

**Published:** 2023-09-06

**Authors:** Paloma Hernández-Muñiz, Celia Borrero, Javier Ordóñez-Martín, Ana M. Pastrana, Manuel Avilés

**Affiliations:** Departamento de Agronomía E.T.S.I.A., Universidad de Sevilla, Ctra. Utrera Km 1, 41013 Seville, Spain

**Keywords:** *Fusarium oxysporum* f. sp. *fragariae*, rice bran, residual strawberry extrudate, fishmeal, disease severity

## Abstract

Anaerobic soil disinfestation (ASD) is proposed as an alternative to the use of chemical fumigants against Fusarium wilt in strawberry crops. Different residual wastes (rice bran, fishmeal, and residual strawberry extrudate) were assessed as amendments for ASD. Two different concentrations and two incubation durations were tested in growth chamber trials. The abundance of several microbial groups was noted before and after the treatments. Strawberry plants were grown in the treated soils to record Fusarium wilt disease severity. The population density of *F. oxysporum* increased after ASD in most amendments with rice bran and residual strawberry extrudate. Changes in *Trichoderma* spp., copiotrophic bacteria, and *Streptomyces* spp. populations were observed after anaerobiosis treatments and plant trials. A reduction in the disease severity was achieved in ASD-treated soils with 20 t/ha of rice bran at both 25 and 60 days of incubation, but not when using a 13.5 t/ha dose. Similarly, treatments using 19.3 t/ha of fishmeal for both incubation durations were able to reduce disease severity. In contrast, a severity reduction was only obtained in soils treated with 25.02 t/ha of the residual strawberry extrudate and incubated for 60 days in anaerobic conditions. Two of the three by-products tested were able to reduce Fusarium wilt symptoms in strawberry plants after an ASD-treatment period of only 25 days. Accordingly, the technique seems promising for strawberry growers in Huelva, Spain, and highly sustainable by giving value to residues produced in surrounding areas.

## 1. Introduction

Strawberry (*Fragaria* x *ananassa* Duch.) is one of the biggest commodities in the province of Huelva, Spain, where 98% of the national production takes place [1]. Historically, the management of soilborne diseases affecting this crop has been highly dependent on the application of chemical fumigants such as 1,3-dichloropropene, metam sodium, dazomet, chloropicrin, and some of their mixes. However, in Spain, soil diseases resurged after following the European legislation on the use of pesticides (EC Regulation 1107/2009) [2] which restricts the use of the main chemical fumigants in Europe. In Huelva, a high occurrence of root and crown rot in strawberry caused by *Macrophomina phaseolina* (Tassi) Goid has been reported [3]. Symptoms of Fusarium wilt have also been detected, associated with *Fusarium oxysporum* f. sp. *fragariae* (*Fof*) (Sacc.) W.C. Synder & H. N. Hans [4] and *F. proliferatum* Schltdl [5]. Therefore, it is essential to search for alternative methods to chemical fumigants.

Anaerobic soil disinfestation (ASD) is attracting particular interest among researchers as an alternative to the use of chemical fumigants for the management of soilborne pathogens [6,7]. The technique involves the addition of organic amendments to the soil, irrigation to field capacity, and covering the surface with plastic tarps to achieve reductive conditions [8,9]. Organic amendments should be rich in labile carbon, such as molasses [10], ethanol [11], and rice bran, which have been found to reduce Verticillium wilt in strawberry [12]. Disease suppression due to ASD has been ascribed to changes in soil microbial communities that have been identified after the treatment [13,14]. The ability of some soil microbes to directly antagonize plant pathogenic fungi and compete with them for nutrients [15,16] and available oxygen in soil [17] can contribute to the disinfestation process. In addition, the organic acids released during anaerobic disinfestation could contribute to the effectiveness of the treatment [18]. 

The efficacy of ASD to control soilborne diseases depends on the used carbon source and the dose [19]. In the case of *F. oxysporum* f. sp. *lycopersici*, suppression is known to be related to C:N ratio, temperature, and duration of the ASD treatment [20]. Additionally, the effectiveness of ASD has been demonstrated when oxygen concentration was around 1% or when the soil redox potential was between -300 and 200 mV [21]. Successful reducing *F. oxysporum* populations requires accumulation of more than 300 h at a soil temperature of around 30 °C [22]. Special care must be taken when anaerobic conditions together with organic amendments cannot control Fusarium wilt, as pathogen populations could increase by effectively competing for the organic substrates added to the soil [23]. 

One of the major rice-producing areas in Spain is located less than 100 km from Huelva, making rice bran readily available as an amendment for strawberry growers, and hence it was used as a reference amendment in our study [12]. Fish manufacturing is another source of great value for the Huelva province and fishmeal is a by-product of 45% of the weight of the fish. Fishmeal contains a high amount of oxidizable carbon and nitrogen [24], making it a great amendment for ASD. Strawberry extrudate is another by-product with potential for ASD, due to its high concentration of labile carbon, and it has been recently proven as an effective ASD amendment for the management of the soilborne pathogen *M. phaseolina* in strawberry plants [25]. It is a by-product of the strawberry processing industry and is obtained by processing strawberry puree to generate secondary products such as jams or fruit additions for yogurts. Thus, strawberries are extruded through sieves with different mesh sizes (2.2 mm and 0.6 mm), leaving behind fibrous parts and achenes from the pulp and the liquid part. 

In this study, our main objective was to evaluate different ASD techniques to reduce *Fof* inoculum density in soil and disease severity in strawberry plants. Three by-products available from industries close to Huelva (rice bran, fishmeal, and residual strawberry extrudate) were tested at two different doses (low and high) and two different incubation durations (25 and 60 days). In addition, changes in the density of potential biocontrol microbes (*Trichoderma* spp., copiotrophic bacteria, *Streptomyces* spp., and fluorescent *Pseudomonas* spp.) were quantified after treatments.

## 2. Materials and Methods

The experimental design was divided into two parts. The first part evaluated inoculum reduction after treatments (Section 2.2). In the second part, *F. oxysporum* population densities and the disease severity of the strawberry plants were assessed after the plant trial (Section 2.3).

### 2.1. Soil and Amendment Characterization

#### 2.1.1. Soil Characterization

Three soil samples, from a strawberry field without disease history, were taken from the first 20 cm (removing the 0.5 cm upper layer). Soil characterization was performed by the Agricultural Research Service at the University of Seville, Spain. Soil parameters (Table 1) were determined using the following methods: pH [26], Olsen phosphorous [27], oxidable organic matter and carbon [28], exchange cations [29], available trace elements [30], texture [31], bulk density and field capacity [32], electrical conductivity (conductivity in extract 1/5), and nitrogen and carbon total, which were determined using a LECO CNS-Trumac Elemental Auto-Analyzer (St. Joseph, MI, USA). 

#### 2.1.2. Amendment Characterization 

The substrates for ASD were obtained from commercial sources: rice bran (Arrozúa S.C.A., Seville, Spain), residual strawberry extrudate (Svz S.A.U., Huelva, Spain), and fishmeal from open water fishing and the canning industry (Usisa S.A., Huelva, Spain). The C:N, dry matter ratios, and total sulfur of the amendments were determined by the Agricultural Research Service at the University of Seville, Spain (Table 2). 

Total C, N, and S were established by the Dumas method described in the LECO CNS-Trumac Elemental Auto-Analyzer (St. Joseph, MI, USA). The determination of oxidable organic C was carried out according to the protocol described by Walkley and Black (1934) [28]. Moisture content was assessed according to the Spanish standard UNE-EN 13040: 2008 (Madrid, Spain: AENOR, 2008).

### 2.2. Trials to Evaluate the Efficacy of Different Treatments for Inoculum Reduction

#### 2.2.1. Experimental Design 

Two similar trials at different inoculation levels were conducted. Soil and inoculum mixes were prepared to obtain a concentration of 4 × 10^3^ cfu of *Fof*/g soil for the first trial and 1 × 10^4^ cfu of *Fof*/g soil for the second. The soil inoculum mixes were homogenized and incubated for one week at room temperature for the establishment of the pathogen. At that point, the number of *Fof* colonies was re-estimated with a sequence of 1/10 dilution sets transferred in triplicate to Komada selective medium [33]. Respectively, a concentration of 2 × 10^3^ cfu/g soil and 6 × 10^3^ cfu/g soil was established. 

Each of the two trials had fourteen treatments and three repetitions per treatment. Amendments were made at two different doses, low and high, and anaerobic conditions remained for 25 or 60 days. The amendment dose of each treatment was adjusted so that the oxidizable carbon contents were equivalent to each other. For rice bran, the doses of 13.5 t/ha (4.91 g/kg soil = low dose) and 20 t/ha (7.28 g/kg soil = high dose), already described by Mazzola et al. (2018) [12], were taken as reference. Treatments with fishmeal were amended at 19.3 t/ha (7.02 g/kg soil = low dose) and 28.59 t/ha (10.41 g/kg soil = high dose). For the residual strawberry extrudate, doses of 16.89 t/ha (6.15 g/kg soil = low dose) and 25.02 t/ha (9.11 g/kg soil = high dose) were assayed (Table 3). Furthermore, an unamended control treatment and a chemical treatment with metam sodium 50% *w*/*v* (Raisan-50, Lainco S.A., Barcelona, Spain) at 300 L/ha were included. Fishmeal treatments amended with 28.59 t/ha were eliminated from the study, as these doses were found to be phytotoxic to the strawberry plants.

The experimental unit consisted of a container with an internal volume of 2.5 L lined with a black plastic bag. In each bag, 4.1 kg of inoculated soil was added and brought to field capacity. The amount of water needed to reach field capacity was calculated considering the moisture contents of the soil and the amendments for each treatment. Amendments and water were incorporated into the containers with the help of an automatic homogenizer. Immediately, bags were closed at the top to allow for anaerobic conditions to develop and fitted with a 15 mL falcon tube cut at the bottom and closed with a cap to facilitate redox readings. Both trials were randomly arranged in a growth chamber and incubated in the dark at 33/23 °C. The thermoperiod was chosen to simulate the environmental conditions of the soil in Huelva during the summer months.

#### 2.2.2. Soil Inoculum Preparation

*Fusarium oxysporum* f. sp. *fragariae* strain F.74 isolated in Huelva (Spain) by Borrero et al. (2017) [4] was used to prepare the soil inoculum. The fungus was grown on AMAP medium (agar, malt extract, asparagine, and the fertilizer Peter’s foliar feed 27 + 15 + 12; N + P_2_O_5_ + K_2_O (Scotts, Heerlen, Holanda)) [34] for one week. Shortly thereafter, rice grains were inoculated in polypropylene bags with filters (PPD75/REH/V37-53, SacO2, Deinze, Belgium) according to the procedure described by Benson and Parker (2016) [35]. Once the pathogen had completely colonized the rice grains, they were dried under sterile conditions. Colonized grains were ground with an Ika M20 grinder (IKA-Werke, Staufen, Germany) and sieved through a 0.425 mm aperture sieve. Then, 300 g of the grounded inoculum was mixed with 1 kg of the above-mentioned soil, which was previously sieved and autoclaved on 3 consecutive days for 1 h at 120 °C. The mix was shaken until completely homogenized and incubated at room temperature. After one week, inoculum-soil titration was performed to estimate the final propagule concentration [36]. The estimated soil inoculum concentration was 2 × 10^6^ cfu/g.

#### 2.2.3. Redox and pH Data Collection

Several redox measurements were performed at different times during the ASD treatments: at 24 h, 10 days, 25 days, and 60 days after the start of the treatments. For redox measurements, the SensoLab Benchtop pH/ORP Meter (PM1000) with the ORP1000 Polycarbonate Laboratory ORP Sensor (Sensorex Corporation, Garden Grove, CA, USA) was used. The soil redox potential values were corrected to relate to the redox potential of a standard hydrogen electrode. The ORP reading in mV was adjusted to Eh mV, and the addition of 201 mV was necessary [37]. To calculate the critical redox potential of soil, which is an indicator of reduced conditions, the formula [CEh = 595 mV − 60 mV (soil pH)] was used [38]. The value of 263.6 mV was determined as the threshold below which soil is considered anaerobic at a soil pH of 5.52 (Table 1) [39]. Soil pH was determined immediately after treatments. For that, a sample of 10 g of sieved soil (200- and 2-millimeter mesh) was mixed with 25 mL of Milli-Q quality water, shaken for 30 min at 160 rpm, and centrifuged for 15 min at 2500 rpm. Lastly, pH measurements of the supernatant were taken with a GLP22 Crison pH meter (Hach Lange Spain, S.L.U, Barcelona, Spain).

#### 2.2.4. Microbial Populations

The population densities of four potential biocontrol agents were determined. For the determination of *Trichoderma* spp., fluorescent *Pseudomonas* spp., and copiotrophic bacteria, the method described by Borrero et al. (2004) [40] was followed. Colonies of *Streptomyces* spp. were counted on 1/50 tryptone-soybean medium using the method described by Mazzola et al. (2018) [12]. In addition, the density of *F. oxysporum*/*Fof* (*F. oxysporum* colonies from soil and amendments + *Fof* colonies from inoculum) was determined as described above (Section 2.2.1). For this purpose, a 10-gram sample per experimental unit was taken from the initial soil one week after treatments.

### 2.3. Plant Disease Severity Evaluation after Treatments

#### 2.3.1. Experimental Design

Soils were aerated after treatments for one week. Then, each repetition of the fourteen treatments was transferred to three 0.65 L pots (n = 9 pots per treatment). One bare root strawberry transplant cultivar ‘Rábida FNM’ (FNM S.A., Huelva, Spain) was planted in each pot. Plants were distributed in a growth chamber in three randomized blocks with three repetitions per block and treatment, and maintained for 140 days at 30 °C day and 25 °C night with a photoperiod of 12 h.

#### 2.3.2. Disease Development and Data Collection

Disease severity was assessed considering leaf wilting and crown vascular necrosis as the main symptoms of Fusarium wilt. Weekly observations were taken for disease evolution. Plants were scored with the relative number of symptomatic leaves per plant (number of symptomatic leaves/total number of leaves). Crown symptomatology was measured as a percentage between 0 and 1, with 0 = crown without vascular necrosis, and 1 = crown with complete vascular necrosis. At the end of the pathogenicity trial, petiole pieces (1 cm) were taken and disinfected with 1% sodium hypochlorite for two minutes, then immersed in water for one minute, and finally, placed on Komada’s selective medium [36] to confirm the presence of the inoculated pathogen.

#### 2.3.3. Microbial Populations

The population densities of four potential biocontrol organisms were determined as described in Section 2.2.4. For this purpose, 10-gram samples were taken at the end of the plant trials. Then, a combined sample from the rhizospheres of the plants belonging to the three plots of each block and treatment was analyzed.

### 2.4. Statistical Analysis

Data collected from two trials were analyzed with the software Statgraphics Centurion 18 (18.1.13 version; Statgraphics Technologies, Inc., The Plains, VA, USA). Data from the two trials were pooled for statistical analysis after finding no significant treatment × trial interaction in factorial ANOVA. Treatments, trials, and their interaction were treated as fixed effects, and blocks nested in trial and its interaction were considered as random effects. The variables analyzed were: foliar disease severity and the percentage incidence of crown vascular necrosis (n = 18); cumulated anaerobic conditions and pH in the soil post-treatments (n = 6); and population density of the microbiological groups (*F. oxysporum*/*Fof*, *Trichoderma* spp., fluorescent *Pseudomonas* spp., *Streptomyces* spp., and copiotrophic bacteria) present in the soil post-treatments and at the end of the plant trials (n = 6). When necessary, the data were transformed for compliance with ANOVA requirements and were tested using Levene, Bartlett, and Cochran’s tests. Overall relationships between the different variables studied were evaluated with a regression analysis.

## 3. Results

### 3.1. Physicochemical Measurements 

The 60-day-treatments with rice bran and fishmeal exhibited significantly the highest accumulation of anaerobic conditions measured as Eh mV below 264 mV. Meanwhile, as expected, the treatments with a duration of 25 days accumulated the lowest cumulative anaerobicity. The treatment with residual strawberry extrudate at 16.9 t/ha was the only one with no significant differences in cumulative anaerobicity between incubation durations (Table 4). 

All treatments showed significantly higher pH values than the control treatment. The fishmeal treatments had the maximum pH values. No significant differences in pH were observed between 60 and 25 days of incubation, except for the treatment amended with 13.5 t/ha of rice bran, where the incubation for 60 days reached a significantly higher pH. Dose effects were also observed with this amendment when incubated for 25 days. However, no significant changes in pH were observed between amendment doses or incubation durations in the fishmeal and the residual strawberry extrudate treatments (Table 4).

### 3.2. Population Densities of Fusarium oxysporum/Fusarium oxysporum f. sp. Fragariae

**Figure 1:**
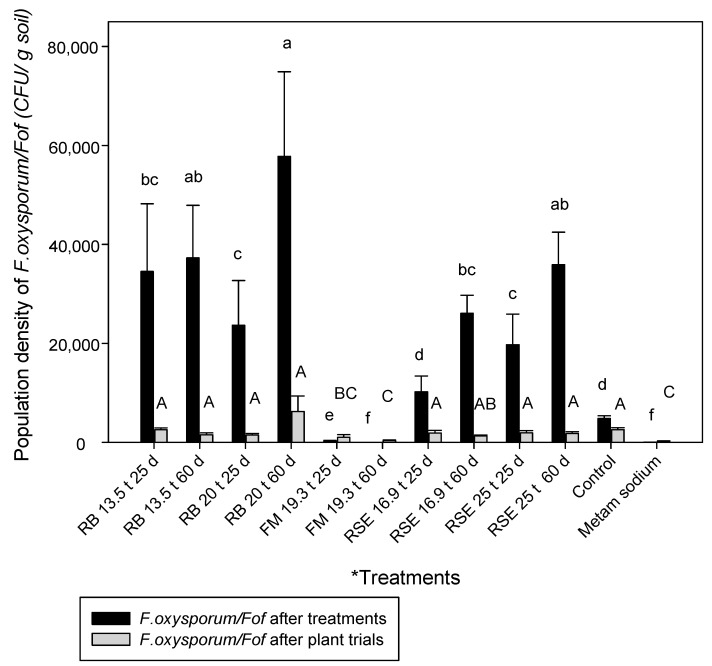
Effects of soil treatment on population densities of *F. oxysporum*/*Fof* after treatments and after the plant trials. The black bars represent the mean values of *F. oxysporum*/*Fof* density post-treatments and the gray bars represent the mean values of *F. oxysporum*/*Fof* density post-plantation. Lines denote the standard errors. * Treatments: RB = rice bran; FM = fishmeal; RSE = residual strawberry extrudate. The first number corresponds to t/ha amended and the second number to the treatment duration (25 or 60 days). Data of cfu/g soil of *F. oxysporum*/*Fof* after treatments and of *F. oxysporum*/*Fof* after plant trials were transformed as x^0.22^ and x^0.25^, respectively. Different letters in the bars indicate significant differences according to ANOVA followed by Duncan’s test (*p* < 0.05), n = 6.

The fishmeal and metam sodium treatments were the only ones that were followed by significantly lower densities of *F. oxysporum*/*Fof* than the control treatment at both durations, after treatments, and after plant trial. On the other hand, after treatments, the population density in the rest of the treatments exceeded the control, except for the one amended with 16.9 t/ha of residual strawberry extrudate for 25 days (Figure 1).

According to the inoculation level, the trial was the only factor with statistical significance in the *F. oxysporum*/*Fof* population density after treatments.

### 3.3. Population Densities of Potential Biocontrol Microbes

After the treatments

No colonies of *Trichoderma* spp. were detected in the fishmeal treatments. The rest of the treatments had similar population densities as the control, except for the rice bran treatments with short treatment duration (25 days), which presented a significantly higher *Trichoderma* spp. population density (Table 4). 

The number of *Streptomyces* spp. CFUs was consistently lower across all disinfection treatments compared to the control group. However, an exception was observed in the case of the amended treatment involving 16.9 t/ha of residual strawberry extrudate and 25 days of anaerobiosis, where the population density matched that of the control group (Table 4).

When compared with the control, higher densities of copiotrophic bacteria were registered in the treatment amended with 20 t/ha of rice bran and 25 days of incubation duration and the metam sodium. The rest of the treatments exhibited the same population density as the control, except for the residual strawberry extrudate treatment with 60 days of anaerobiosis, for which density population values were lower (Table 4). No colonies of fluorescent *Pseudomonas* spp. were found.

After the plant trials

The only treatments with significantly higher CFU/g of soil of *Trichoderma* spp. than the control treatment were the ones amended with fishmeal and metam sodium. The rest of the treatments displayed a population density similar to the control (Table 5). 

All treatments exhibited the same *Streptomyces* spp. population density as the control except for those amended with 20 t/ha of rice bran and the 60-day treatment amended with fishmeal, which showed significantly lower levels (Table 5).

In regards to the copiotrophic bacteria population, the treatments amended with rice bran at 20 t/ha were the only treatments that showed a significant increase (Table 5).

Finally, the population density of fluorescent *Pseudomonas* spp. was calculated, but no significant differences were observed between treatments (data not shown). 

### 3.4. Foliar Disease Severity

**Figure 2:**
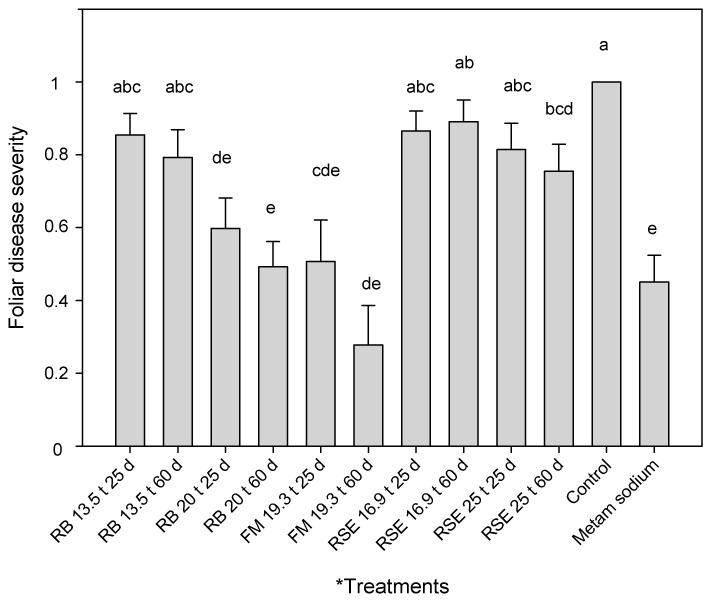
Effect of soil treatments on foliar disease severity (Fusarium wilt) in strawberry plants. Disease measures were taken as percentages of symptomatic leaves per plant (number of symptomatic leaves/total number of leaves), with 0 = healthy plant and 1 = dead plant. The bars represent the mean values of foliar disease severities, and the lines are the standard errors. * Treatments: RB = rice bran; FM = fishmeal; RSE = residual strawberry extrudate. The first number corresponds to t/ha amended and the second number to the treatment duration (25 or 60 days). Data were transformed for statistical analysis as x^4^. Different letters in the bars indicate significant differences according to ANOVA followed by Duncan’s test (*p* < 0.05), n = 18.

Treatments amended with rice bran at 20 t/ha and the fishmeal amended treatments showed no significant differences with metam sodium. These treatments also had significantly lower foliar severities than the control. Additionally, the treatment amended with 25 t/ha of the residual strawberry extrudate and incubated for 60 days also registered lower foliar disease severity than the control (Figure 2). A significant effect of the amendment dose was observed for the treatments using rice bran, and the ones using 13.5 t/ha exhibited higher disease severity than the ones using 20 t/ha.

Furthermore, significant differences between trials were observed. Higher foliar disease severity was recorded in the trial inoculated at the higher dose (1 × 10^4^ CFU/g soil), but the treatments × trial interaction was not significant in the factorial ANOVA.

### 3.5. Vascular Disease Severity

**Figure 3:**
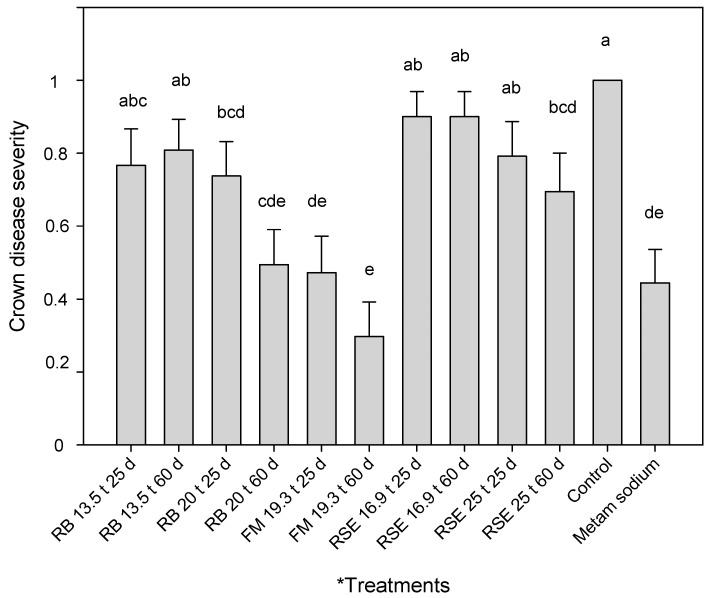
Effect of soil treatments on crown disease severity (Fusarium wilt) in strawberry plants. Disease measures were taken as percentages, being 0 = healthy crown and 1 = crown with complete vascular necrosis. The bars represent the mean values and lines denote the standard errors. * Treatments: RB = rice bran; FM = fishmeal; RSE = residual strawberry extrudate. The first number corresponds to t/ha amended and the second number to the treatment duration (25 or 60 days). Different letters in the bars indicate significant differences according to ANOVA followed by Duncan’s test *(p* < 0.05), n = 18.

The treatments that showed lower crown vascular necrosis than the control were the ones amended with rice bran at 20 t/ha, the treatments amended with fishmeal, and the treatment amended with 25 t/ha of the residual strawberry extrudate and incubated for 60 days. All these treatments showed no significant differences concerning metam sodium treatment (Figure 3). 

Similarly, as with foliar disease severity, the trial factor was significant, and higher crown disease severity values were recorded in the trial inoculated at a higher dose (1 × 10^4^ CFU/g soil), but the treatment × trial interaction was not significant in the factorial ANOVA.

### 3.6. Correlations

There were negative correlations between foliar disease severity and pH values, cumulative Eh mV h below 264 mV, and CFU/g of soil of *Trichoderma* spp. post-plantation CFU/g of soil. However, positive correlations were found between foliar disease severity and the population density of *F. oxysporum*/*Fof* post-treatment and post-plantation and crown disease severity (Table 6).

## 4. Discussion

Given these results, the use of local waste (rice bran, fishmeal, or residual strawberry extrudate) for the ASD technique could be an environmentally friendly way to dispose of these residues and to control possible diseases in strawberry production. The success of any of these amendments could be an alternative to the use of chemical fumigants and could be a great contribution to the circular economy in the area. 

This research indicated that the use of all proposed amendments, with appropriate duration and dose, was effective in reducing Fusarium wilt severity. Higher doses of both rice bran and residual strawberry extrudate were found to be necessary to control the disease. This would mean removing large quantities of these residues from the system and giving them a second use. For fishmeal, the dose of 19.3 t/ha was found to be effective for disease control. It should be noted that both rice bran and fishmeal at the aforementioned doses have the same disease control capacity at 25 and 60 days. This would mean a reduction in disinfestation duration for the grower before the strawberry plantation. In contrast, the strawberry extrudate needs higher doses and 60 days of anaerobicity to be effective.

The use of soil from the agricultural strawberry area as a potting media, and the implementation of thermoperiod and photoperiod conditions similar to those found in Huelva, mean that the results of this research can be used as a guide for subsequent field trials.

According to Yonemoto et al. (2006) [22], accumulation in the soil for about 280 to 300 h at a temperature of 30 °C should be sufficient to reduce pathogen population. The used thermoperiod conditions (33/23 °C) were chosen to be similar to those occurring in Huelva soils during summer (July and August), when the ASD treatment would take place. Agreeing with the results obtained in this study, the use of these amendments for ASD, with the correct dose and duration, would be suitable for reducing the severity of Fusarium wilt at the indicated temperature. 

Several processes, which are not fully elucidated yet, are involved in the effectiveness of ASD for the control of Fusarium wilt. Some authors described that at least 100,000 mV hours of accumulated anaerobic conditions in the soil was enough to have a significant population reduction in *Fof* [23]. In this study, a reduction in the *F. oxysporum* populations was not always achieved above the 100,000 mV threshold and only occurred in the fishmeal amended treatments (both exceeded the 100,000 mV). This fact indicates the existence of other factors involved in the process.

Soil suppressiveness can be enhanced by adjustment of soil pH at around 7.0, which has been shown to reduce symptoms of Fusarium wilt in several crops, including strawberry [41,42,43,44]. Therefore, the notable increase in pH after the different treatments may have favored disease management in some cases. This statement is consistent with the negative correlation obtained between pH and disease severity. The rise in pH could be due to the consumption of H^+^ through denitrification and the reduction in Fe^3+^, Mn^3+^, and SO_4_^2^ under anaerobic conditions [45]. These results are supported by those obtained by Zhu et al. (2022) [46], which also show a rise in pH concerning the initial soil after treatments. However, these results are opposed to other studies where a reduction in soil pH after the incubation of the amendment is observed [18]. 

The higher pH values in the fishmeal amended treatments could be due to their C:N ratio of 3:1. Low C:N ratios in soil indicate a higher nitrogen accumulation and consequently a higher release of ammonium into the medium [47]. A recent and unpublished study conducted by our group describes the presence of three-times higher ammonium concentration in soils treated with fishmeal than soils treated with rice bran and residual strawberry extrudate. Likewise, research by Serrano (2015) [24] highlighted the inhibitory effect on microbial growth (fungicidal effect) caused by an accumulation of free ammonia generated by a high pH. Accordingly, the population of *F. oxysporum*/*Fof* was reduced after treatment and after the plant trials when using fishmeal as amendment. Furthermore, in the fishmeal treatments, the same fungicidal capacity as metam sodium was observed. 

Shrestha et al. (2021) [20] successfully applied the ASD technique with amendments with C:N ratios between 10 and 40:1 for the suppression of *F. oxysporum* f. sp. *lycopersici* in soils with temperatures above 25 °C. In this study, the amendments with higher C:N content were rice bran and residual strawberry extrudate (C:N = 20:1), and they were less effective in reducing *F. oxysporum* populations than the one with a lower ratio (C:N = 3:1). In addition, in some of the treatments amended with these residues, an increase in *F. oxysporum* populations was recorded. Previous authors reported an increase in *Fusarium* populations when amendments such as rice bran or mustard seeds were added [10,12,39]. The rise in *F. oxysporum* propagules could be explained by the fact that the carbon groups not consumed during treatment would enable the fungus to proliferate. It is known that pathogenic and non-pathogenic forms of *F. oxysporum* are competitive saprophytes, so limited substrate availability at the end of the anaerobic period is essential [6]. Therefore, a balance between the preference of *F. oxysporum* for a specific amendment and the need for carbon input to generate anaerobic conditions during ASD is required for good disinfestation.

A similar trend was observed for *Trichoderma* spp. In the fishmeal amended treatments, no colonies of *Trichoderma* spp. were found after the use of this amendment. However, an important increase in its population was reported after the plant trial. In this case, it is possible that the colonies that were not detected took advantage of the disturbance of soil microbial communities generated by the disinfestation. The increase in the population density of *Trichoderma* spp. could be contributing to disease control, due to the ability of this species to act as a biological control agent against pathogens [48]. Furthermore, a negative correlation was found between the population density of *Trichoderma* spp. after the plant trial and the foliar disease severity. These results are supported by Pastrana et al. (2016) [49], who described the ability of different *Trichoderma* spp. to control strawberry root rot diseases caused by *F. solani* or *M. phaseolina*. 

The capacity of some microorganisms such as *Streptomyces* spp. [50], fluorescent *Pseudomonas* spp., and copiotrophic bacteria [51] to control fungal pathogens is well known. Our results show population changes in these groups of microorganisms after the application of ASD and subsequent plant trials. An increase in the population of copiotrophic bacteria was observed when amended with 20 t/ha of rice bran. This could have contributed to the management of Fusarium wilt. In the case of *Streptomyces* spp., no changes in the population density were observed between the ASD treatments and the control. Likewise, no significant differences were found in *Pseudomonas* spp. populations after the plant trial.

Although the population density of *F. oxysporum*/*Fof* was only reduced in soils treated with fishmeal, the rest of the amendments were also able to reduce Fusarium wilt disease severity when used at the correct dose and duration. Using rice bran at 20 t/ha for a 25-day treatment, it was possible to reduce foliar and crown disease severity by 40% and 30%, respectively. When the duration of the treatment was 60 days, the disease severity was reduced by 50% in both cases. High plant mortality was observed when using a high dose of fishmeal (28.6 t/ha). For this reason, the use of this amendment at high doses was eliminated from the trial and the statistical analysis. However, when using this amendment at 19.3 t/ha and 25 days of incubation, it was possible to reduce foliar and crown disease severity by around 50%. Meanwhile, an average of 72% foliar and crown disease reduction was achieved with 60 days of incubation. Our ongoing studies are evaluating which quantity of residual ammonium in the soil would be desirable to adjust the amendment dose to avoid phytotoxicity problems for future experiments. The use of residual strawberry extrudate as an amendment for ASD allowed a reduction in Fusarium wilt foliar and crown disease severity by 25 and 31%, respectively, when amended at 25 t/ha and incubated for 60 days. In agreement, this amendment has been already reported as an effective ASD amendment in reducing soil propagules and foliar disease severity caused by *Macrophomina phaseolina* in strawberry [25]. 

In summary, the use of ASD with the appropriate duration and concentration of any of these amendments would give the farmer an effective alternative to the use of soil fumigants and, in addition, would promote the circular economy of neighboring sectors. The next steps would include the most successful treatments in real field conditions, where soil and environmental factors will be more heterogeneous.

## Figures and Tables

**Table 1:** Physicochemical parameters of the soil used in this study.

Parameters	Values ^+^
pH (extractor 1/ 2.5 W/V)	5.52 ± 0.06
Electrical conductivity (µS/cm) (extractor 1/5 W/V)	216.20 ± 13.27
Olsen P (mg/kg)	62.18 ± 1.75
Oxidable organic matter (%)	0.42 ± 0.01
Oxidable organic carbon (%)	0.24 ± 0.00
Total N (%)	0.03 ± 0.01
Exchange cations (soluble in ammonium acetate 1N pH 7)	
Ca (cmolc/kg)	1.17 ± 0.09
Mg (cmolc/kg)	0.37 ± 0.02
K (cmolc/kg)	0.52 ± 0.03
Na (cmolc/kg)	0.18 ± 0.01
Available trace elements (soluble in DTPA-TEA-CaCl_2_)	
Fe (mg/kg)	49.92 ± 1.25
Mn (mg/kg)	11.33 ± 0.53
Zn (mg/kg)	4.23 ± 0.12
Cu (mg/kg)	2.73 ± 0.03
Texture:	
Silt (%)	7.18 ± 0.08
Clay (%)	3.59 ± 0.10
Sand (%)	89.24 ± 0.06

^+^ Data represent mean ± standard error.

**Table 2:** Determination of oxidable and total organic carbon, total nitrogen, total sulfur, and the humidity of used amendments.

Amendment	Oxidable Organic Carbon (%)	Total C (%)	Total N (%)	Total S (%)	Humidity (%)
Rice bran	46.51	50.19	2.21	0.24	4.51
Fishmeal	32.53	42.23	10.71	0.93	2.91
Residual strawberry extrudate	37.18	52.37	1.95	0.18	5.42

**Table 3:** Explicative table of the treatments assayed.

Treatments ^a^	Amendment	Doses (t/ha)	Amendment Dose in Soil (g/kg Dry Soil)	Duration (Days)
RB 13.5 t_25 d	Rice bran	13.5 (low)	4.91	25
RB 13.5 t_60 d	Rice bran	13.5 (low)	4.91	60
RB 20 t_25 d	Rice bran	20 (high)	7.28	25
RB 20 t_60 d	Rice bran	20 (high)	7.28	60
FM 19.3 t_25 d	Fishmeal	19.3 (low)	7.02	25
FM 19.3 t_60 d	Fishmeal	19.3 (low)	7.02	60
RSE 16.9 t_25 d	Residual strawberry extrudate	16.89 (low)	6.15	25
RSE 16.9 t_60 d	Residual strawberry extrudate	16.89 (low)	6.15	60
RSE 25 t_25 d	Residual strawberry extrudate	25.02 (high)	9.11	25
RSE 25 t_ 60 d	Residual strawberry extrudate	25.02 (high)	9.11	60
Control	No amendment	-	-	25
Metam sodium	Metam sodium 50% *w*/*v*	-	-	15

Treatments ^a^: RB = rice bran; FM = fishmeal; RSE = residual strawberry extrudate. The first number corresponds to t/ha amended and the second number to the treatment duration (25 or 60 days).

**Table 4:** Effect of treatments on cumulated anaerobic conditions and pH at the end of the treatments.

Treatments ^a^	Cumulative Eh mV h below 264 mV ^b^	pH
RB 13.5 t_25 d	80,997 ± 9900 e	7.09 ± 0.2 c
RB 13.5 t_60 d	285,640 ± 25,290 ab	7.81 ± 0.27 b
RB 20 t_25 d	134,022 ± 9395 d	7.71 ± 0.01 b
RB 20 t_60 d	275,883 ± 13,634 ab	7.83 ± 0.05 b
FM 19.3 t_25 d	191,989 ± 17,854 cd	8.9 ± 0.07 a
FM 19.3 t_60 d	362,954 ± 29,539 a	8.74 ± 0.02 a
RSE 16.9 t_25 d	174,710 ± 17,604 cd	7.03 ± 0.16 c
RSE 16.9 t_60 d	239,404 ± 31,187 bc	6.91 ± 0.12 cd
RSE 25 t_25 d	137,221 ± 18,947 d	6.99 ± 0.13 cd
RSE 25 t_ 60 d	256,560 ± 42,484 bc	6.71 ± 0.11 d
Control	-	6.02 ± 0.15 e
Metam sodium	-	6.88 ± 0.09 cd

Data represent the mean ± standard error. Values followed by different letters indicate significant differences according to ANOVA and Duncan’s test (*p* < 0.05), n = 6 for cumulative Eh mV h below 264 mV and pH variables. Treatments ^a^: RB = rice bran; FM = fishmeal; RSE = residual strawberry extrudate. The first number corresponds to t/ha amended and the second number to the treatment duration (25 or 60 days). ^b^ Cumulative Eh mVh below 264 mV: 201 mV was added to modify the ORP reading from mV to Eh mV. To calculate the threshold value of 263.6 mV, below which anaerobiosis is considered, the formula [Ech = 595 mV − 60 mV (soil pH)] was used. Data for cumulative Eh mV h below 264 mV were transformed as x^0.2^ for statistical analysis.

**Table 5:** Effect of treatments and plant trials in the population densities of potential biocontrol microbes.

Treatments ^a^	*Trichoderma* spp. after Treatments ^b^	*Trichoderma* spp. after Plant Trials ^c^	*Streptomyces* spp. after Treatments ^d^	*Streptomyces* spp. after Plant Trials ^e^	Copiotrophic Bacteria after Treatments ^f^	Copiotrophic Bacteria after Plant Trials ^g^
	×10^3^ CFU/g Soil		×10^4^ CFU/g Soil		×10^7^ CFU/g Soil	
RB 13.5 t_25 d	17.50 ± 5.48 a	3.16 ± 1.32 cd	27.86 ± 13.80 cde	6.82 ± 1.85 abcd	7.55 ± 2.75 abc	1.25 ± 0.28 b
RB 13.5 t_60 d	1.3 ± 0.60 e	2.63 ± 0.64 cd	6.35 ± 1.64 f	4.89 ± 1.11 abcd	1.28 ± 0.18 def	1.02 ± 0.01 bc
RB 20 t_25 d	16.61 ± 5.45 ab	6.90 ± 2.57 cd	19.35 ± 7.17 ef	5.53 ± 3.10 bcd	13.45 ± 3.76 a	2.63 ± 0.46 a
RB 20 t_60 d	8.73 ± 3.34 bc	3.15 ± 1.83 cd	12.12 ± 4.879 ef	2.03 ± 1.45 d	1.75 ± 0.24 cde	2.93 ± 0.43 a
FM 19.3 t_25 d	0 ± 0 f	48.33 ± 3.16 a	7.79 ± 2.71 f	5.56 ± 2.10 abcd	11.49 ± 4.94 abc	1.20 ± 0.2 b
FM 19.3 t_60 d	0 ± 0 f	8.62 ± 3.21 bc	8.70 ± 3.06 f	3.77 ± 1.83 cd	1.30 ± 0.24 ef	0.86 ± 0.3 bc
RSE 16.9 t_25 d	4.25 ± 1.01 cd	5.59 ± 2.68 cd	55.93 ± 12.10 ab	7.55 ± 2.02 abc	2.08 ± 0.45 bcde	0.99 ± 0.3 bc
RSE 16.9 t_60 d	2.29 ± 0.78 de	0.83 ± 0.49 d	8.22 ± 2.79 f	7.28 ± 2.69 abcd	0.84 ± 0.18 g	0.81 ± 0.16 bc
RSE 25 t_25 d	2.64 ± 0.46 cde	4.76 ± 1.51 cd	38.47 ± 8.97 bcd	5.28 ± 2.14 abcd	2.73 ± 0.47 bcd	1.07 ± 0.2 bc
RSE 25 t_ 60 d	1.43 ± 0.68 de	1.64 ± 0.60 d	16.07 ± 4.56 def	9.90 ± 2.44 ab	1.00 ± 0.31 fg	1.04 ± 0.08 bc
Control	2.66 ± 0.59 cde	2.10 ± 0.85 d	91.33 ± 6.80 a	15.74 ± 5.47 a	1.89 ± 0.33 cde	0.57 ± 0.09 bc
Metam sodium	1.95 ± 0.77 e	25.69 ± 8.59 b	47.90 ± 16.60 bc	5.84 ± 1.95 abcd	6.85 ± 1.96 ab	0.49 ± 0.19 c

Data represent the mean ± standard error. Values with different letters mean significant differences (*p* < 0.05) according to Duncan’s test. Treatments ^a^: RB = rice bran; FM = fishmeal; RSE = residual strawberry extrudate. The first number corresponds to the number of t/ha amended and the second number to the treatment duration (25 or 60 days). Data of CFU/g soil of *Trichoderma* spp. ^b^, *Streptomyces* spp. ^d^ and copiotrophic bacteria ^f^ after treatments, and *Trichoderma* spp. ^c^ *Streptomyces* spp. ^e^ and copiotrophic bacteria ^g^ after plant trials were transformed for statistical analysis as x^0.3^, x^0.3^, x^-0.4^, x^0.4^, x^0.5^, and x^0.7^, respectively. n = 6 for all variables.

**Table 6:** Correlations between different measurements of the trials inoculated with *Fusarium oxysporum* f. sp. *fragariae*/*Fof*.

Parameters	r^2^	P ^a^	Equation
Foliar disease severity ^a^–pH ^b^	0.28	0.0000	Foliar disease severity = 1.93758 − 0.169673 × pH
Foliar disease severity–Cumulative Eh mV h below 264 mV ^c^	0.14	0.0020	Foliar disease severity= (0.901166 − 1.60402^−12^ × Cumulative Eh mV h^2^)^2^
Foliar disease severity–*Trichoderma* spp. after plant trial ^d^ cfu density	0.29	0.0000	Foliar disease severity = (0.836533 − 5.55082^−11^ × *Trichoderma* spp. after plant trial^2^)^2^
Foliar disease severity–*F. oxysporum*/*Fof* after treatments ^e^ cfu density	0.23	0.0000	*F. oxysporum*/*Fof* after treatments = (10.2173 + 156.004 × foliar disease severity)^2^
Foliar disease severity–*F. oxysporum*/*Fof* after plant trial ^f^ cfu density	0.10	0.0057	Foliar disease severity = 0.548807 + 0.00330571 × √(*F. oxysporum*/*Fof* after plant trial)
Foliar disease severity–Crown disease severity ^g^	0.76	0.0000	Foliar disease severity = √(0.0918238 + 0.81964 × Crown disease severity^2^)

The foliar disease severity ^a^ was correlated with the pH ^b^ values measured just after the end of the treatments, cumulative Eh mV h below 264 mV ^c^, *Trichoderma* spp. Post-plantation ^d^, *F. oxysporum* population density one week after treatments ^e^, and with the values measured one week after the plant trials ^f^ and crown disease severity ^g^.

## Data Availability

The data presented in this study are available on request from the corresponding author.
